# Genome-wide analyses of the *Bemisia tabaci* species complex reveal contrasting patterns of admixture and complex demographic histories

**DOI:** 10.1371/journal.pone.0190555

**Published:** 2018-01-24

**Authors:** S. Elfekih, P. Etter, W. T. Tay, M. Fumagalli, K. Gordon, E. Johnson, P. De Barro

**Affiliations:** 1 CSIRO, Black Mountain Laboratories, ACT, Australia; 2 Department of Zoology, University of Cambridge, Cambridge, United Kingdom; 3 Institute of Molecular Biology, University of Oregon, Eugene, OR, United States of America; 4 Department of Life Sciences, Silwood Park campus, Imperial College London, Ascot, United Kingdom; 5 CSIRO Ecosciences Precinct, Brisbane, QLD, Australia; Agricultural Research Organization Volcani Center, ISRAEL

## Abstract

Once considered a single species, the whitefly, *Bemisia tabaci*, is a complex of numerous morphologically indistinguishable species. Within the last three decades, two of its members (MED and MEAM1) have become some of the world's most damaging agricultural pests invading countries across Europe, Africa, Asia and the Americas and affecting a vast range of agriculturally important food and fiber crops through both feeding-related damage and the transmission of numerous plant viruses. For some time now, researchers have relied on a single mitochondrial gene and/or a handful of nuclear markers to study this species complex. Here, we move beyond this by using 38,041 genome-wide Single Nucleotide Polymorphisms, and show that the two invasive members of the complex are closely related species with signatures of introgression with a third species (IO). Gene flow patterns were traced between contemporary invasive populations within MED and MEAM1 species and these were best explained by recent international trade. These findings have profound implications for delineating the *B*. *tabaci* species status and will impact quarantine measures and future management strategies of this global pest.

## Introduction

Species invasions are major drivers for declines in species richness [1] and have arisen to prominence as major threats to the social and economic well-being of communities [2–4]. More than 120,000 species have invaded Australia, Brazil, India, South Africa, the United States of America and the United Kingdom [5], with management costs estimated at US$314 billion annually [6,7]. The features that make species invasive are diverse and idiosyncratic, but one element that is consistently important for an invading species is the ability to adapt rapidly to environmental change [8–10]. When such adaptation is genetic, then evidence for it can be traced by comparing the genomes of invasive species and non-invasive ones.

To address this question, we use the whitefly, *Bemisia tabaci*, as it contains some of the world’s most damaging agricultural pests as well as species that show no invasive capacity [11]. This complex therefore presents a compelling model for comparing closely related invasive and non-invasive species.

The relatedness of different members of the *B*. *tabaci* complex has been previously characterized [12]. Based on mitochondrial DNA markers (mtCOI), there are four major geographically defined clades: (I) Sub-Saharan Africa, (II) New World, (III) Asia, and (IV) Africa/Middle East/Asia Minor/Central Asia/Mediterranean. The latter contains four putative species. Three of them, Middle East-Asia Minor 1 (hereon MEAM1; referred to in the older literature as biotype B), Middle East-Asia Minor 2 (hereon MEAM2), and Mediterranean (hereon MED; referred to in the older literature as biotypes Q, J and L) have become globally invasive whereas the fourth, Indian Ocean (hereon IO) has not [13–15]. IO is found in several Indian Ocean islands and parts of East Africa [13]. MEAM1 has invaded well beyond its presumed home range that extends across the region encompassing Iran, Israel, Jordan, Kuwait, Pakistan, Saudi Arabia, Syria, United Arab Emirates and Yemen, to more than 50 countries across, Europe, Asia, Africa and the New World [16]. MED has a more complex home range that extends across West Africa and the counties bordering the Mediterranean Basin (e.g., Algeria, Crete, Egypt, France, Greece, Israel, Italy, Morocco, Portugal, Spain, Sudan, Syria and Turkey) [16]. It has spread to countries in Asia, the New World and parts of Africa. MEAM2 was for a long time known only from the island of Reunion, but has more recently been detected in Iraq (GenBank KX679576; sample collected in 2005), Turkey, Peru and Japan [17,18]. Investigating the evolutionary genetics of *B*. *tabaci* has largely been confined to the use of mtCOI or a small number of microsatellites [19,20, 21] which, together with a highly repetitive genome (~680–690 Mb) [22,23, 24], has limited our ability to gain an in-depth understanding of its diversity and demographic history. These limitations are rapidly being bypassed by next-generation sequencing (NGS) methods [25, 26, 27, 28]. For instance, the Restriction Associated DNA- tags sequencing (RADseq) protocol provides opportunities to sample the genome, in non-model organisms with limited genomic information [29–34]. In insects, RADseq has been used to address biological questions on demography and dispersal of invasive insect pests [35–38], patterns of gene flow, phylogeography and species delimitation [39–42].

The application of RADseq, despite its great potential for single nucleotide polymorphism (SNP) discovery and generating thousands to millions of informative markers across the genome, may be affected by several biases such as PCR artefacts, false genotyping due to low sequencing depth [43], and ascertainment bias introduced by polymorphisms that may occur at restriction sites [44]. It also requires both high quality and quantity of genomic DNA. This latter requirement for library preparation is one of the most important shared limitations of RADseq [45], and is an important limiting factor for studying organisms with small body size like whiteflies.

Recently, a genotyping-by-sequencing variant protocol that requires low input DNA, Nextera-tagmented reductively amplified DNA (nextRAD) [46–48], has been developed. In this protocol, the Nextera kit (Illumina, Inc.) is used to tagment genomic DNA via *in vitro* transposition and attach short adaptors. A PCR step is then performed with primers that bind to adapters with selective sequences; thereby amplifying only fragments terminating in these selective sequences. This protocol generates RAD-like data (reads pile up at particular loci across the genome) without the use of restriction enzyme digests. Unlike the earlier methods, it requires much lower quantities of input DNA, making it possible to obtain genome-wide information from single individuals of non-model organisms with unknown or complex genome structure and small body size. *B*. *tabaci* is such a species with an adult body size of typically 1~2 mm. Using nextRAD, a variant RADseq protocol, we explore global gene flow patterns, population structure, demographic history, signatures of interspecific hybridization and species divergence in whiteflies using field-collected individual male samples from both invasive and non-invasive species.

## Material and methods

### Sample collection

**Fig 1:**
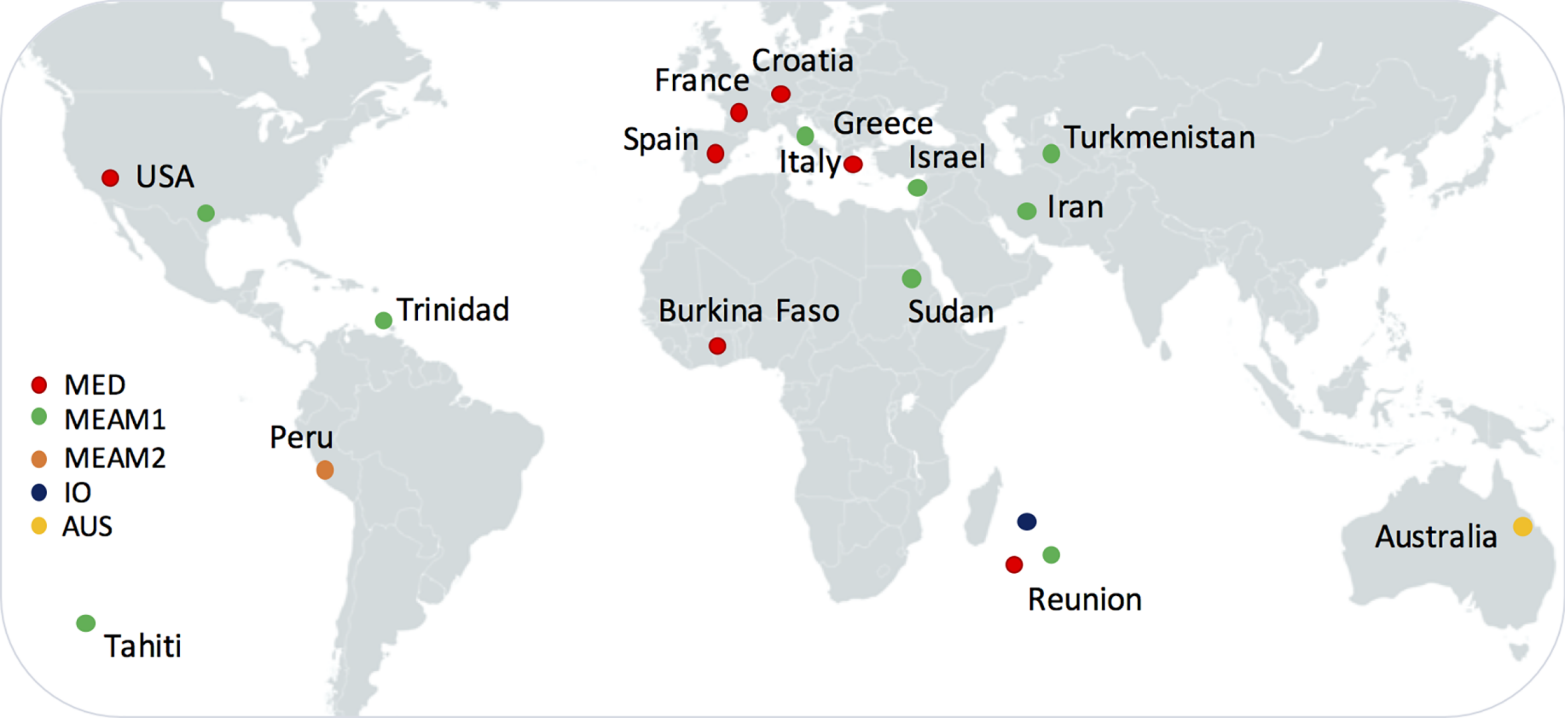
Map showing the sampling locations of the *B*. *tabaci* species (species status initially determined using mtCOI genotyping and further confirmed by genome-wide SNPs). Details of sampling size and exact locations are listed in S1 Table.

Individual specimens of MED, MEAM1, MEAM2, IO and AUS (a member of the complex from Australia that belongs to the Asia clade) were collected between 2006 and 2013 from 17 countries (Fig 1), the Americas [USA (Arizona and Texas), Peru, Trinidad], Europe (Croatia, Cyprus, France, Greece, Italy, Spain), Oceania [French Polynesia (Tahiti), Australia (Queensland)], Africa/Indian Ocean (Burkina Faso, Sudan, Réunion Island) and the Middle-East (Iran, Israel and Turkmenistan) (Fig 1, S1 Table). Specimens were preserved in 95% ethanol. No specific permissions were required for the locations where insect samples were collected. Sampling collections did not involve endangered or protected species.

### DNA extraction, nextRAD sequencing

Total genomic DNA (gDNA) was extracted from each individual male whitefly sample using the DNeasy blood and tissue Kit (Qiagen, Valencia, CA) that also included an RNase treatment step as recommended by the manufacturer. Extracted gDNA samples were eluted in 20 μl AE buffer and quantified using the Qubit 2.0 fluorometer (Life Technologies, Carlsbad, CA). A total of 95 *B*. *tabaci* specimens, each with an approximately 30 ng to 40 ng gDNA yield, were selected for nextRAD genotyping. An amount of 18.0 μl of each sample was dried down in a Speedvac concentrator and resuspended in nuclease-free water at 1.5 ng/μl. A few samples had less than 5ng total and were thus resuspended in 5 μl.

Species identity was based on mtCOI fragment (~657 bp), BLAST search against the *Bemisia* mtCOI database available on GenBank. All haplotypes reported in this study were submitted to GenBank and Accession numbers are available in S1 Table. The extracted gDNA was used to prepare nextRAD libraries following the protocol which uses selective PCR primers to amplify genomic loci consistently between samples [46].

First, gDNA (6ng or less depending upon extraction yield) was fragmented with Nextera reagent (Illumina Inc.), which also ligates short adapter sequences to the ends of the fragments. Fragmented DNA was then amplified, with one of the primers matching the adapter and extending 9 arbitrary nucleotides into the genomic DNA with the selective sequence. Thus, only fragments starting with a sequence that can be hybridized by the selective sequence of the primer will be efficiently amplified. The resulting fragments are fixed at the selective end, and have random lengths depending on the initial Nextera fragmentation. For these reasons, amplified DNA from a particular locus is present at many different sizes and careful size selection of the library is not needed. For this study, an arbitrary 9-mer was chosen from those previously validated in the lab in smaller genomes, which didn’t appear to target repeat-masked regions in publically available insect genomes and that would approximate the results of standard RAD sequencing projects using the restriction endonuclease *Sbf*I [30, 31].

### Data filtering

The quality of the fastq sequences was assessed using FastQC (http://www.bioinformatics.babraham.ac.uk/projects/fastqc) which provides a report on quality scores per sequence, N content, GC content and sequence duplication levels. Based on these reports, a trimming by quality (Phred quality score < 20), to a length of 101 bp, was done in Trimmomatic [49].

Given that *Bemisia tabaci* harbors a wide range of endosymbionts, it was crucial to evaluate the proportion of reads corresponding to our target organism. A total number of 1000 high-quality reads were shuffled, randomly selected from each sample and were used for a BLASTN search against the NCBI sequence database. We retained samples showing more than 50% of their reads mapping to *B*. *tabaci*. The following step was to map the reads in each sample to five of the most important endosymbionts in the *Bemisia* gut, i.e. *Candidatus Portiera aleyrodidarum* (NC_018507), *Candidatus Hamiltonella defensa* (AJLH00000000.2), *Candidatus Cardinium hertigii* (NZ_CBQZ010000011), *Rickettsia* sp. (AJWD00000000), and *Wolbachia sp*. (NC_002978.6). The endosymbiont genomes (accessed from NCBI), were used for read mapping in BWA-MEM [50]. Unmatched sequences, corresponding to the whitefly genome, were fed to the stacks pipeline for subsequent bioinformatics analyses (S3 Table).

### SNP calling

The SNP calling was performed using two approaches. First, we applied *de novo* SNP calling to address species delimitation, phylogeny and possible patterns of introgression. The second approach relied on mapping the nextRAD reads to the *B*. *tabaci* reference genomes available [23, 24]. This analysis aimed at investigating gene flow and migration pathways between populations within the same species. The SNP calling based on mapped reads to the genome involved samples from MED and MEAM1 only since they are the most globally invasive species within the complex.

### *De novo* approach

We first proceeded with a *de novo* approach using the totality of the samples retained after quality filtering (n = 71) regardless of their presumed species status or sampling location. This approach was used to address the species delimitation question and verify whether our analyses are consistent with the Mitochondrial gene-based phylogenies previously reported for this cryptic species complex. The SNP-calling was performed using Stacks (v1.35 [51]). The fastq sequences were de-multiplexed using *process-radtags* implemented in *Stacks*. We first performed a *de novo* SNP calling using *ustacks* to align the short reads into exactly-matching stacks. We used *m = 2* (with *m* being the minimum depth of coverage required to create a stack), the maximum distance (in nucleotides) allowed between stacks value was 2 and the maximum distance allowed to align secondary reads to primary stacks was equal to 4. Then, a catalogue was built using *cstacks*, merging alleles together from all the samples in the dataset. We allowed 2 mismatches between samples to build a stack. The stacks were then compiled into sets that can be searched against the catalogue generated by *cstacks*. The last step in the *Stacks* pipeline (*populations*) generated summary statistics output files including a vcf file, which was fed to VCFtools [52] to extract the genotypes and the read depth per site for every individual sample in the dataset. Given that one of the main aims of this study is the species delimitation of *B*. *tabaci* cryptic complex, we used PyRAD, an additional pipeline developed specifically for RADseq data looking at introgression and phylogenetic inferences. The advantage of this pipeline is that it takes into account the insertions and deletions (Indels) since the clustering process of reads into loci relies on global alignment tools [53].

The filtering step is set to replace base calls with Q < 20 with an ambiguous base (N) and discard any read with more than four Ns. The clustering step of RAD sequences was performed using 85, 88 and 92% rates of clustering similarity. The minimum depth of coverage for a cluster was set at 6X. The three runs returned similar and consistent results, therefore we conducted subsequent analyses using the 85% similarity run.

### Reference mapping

A total number of samples (71) were mapped to the MEAM1 and the MED genomes. We used the Burrows-Wheeler Aligner (BWA) program (v. 0.7.12 [50]), specifically the BWA-MEM algorithm, which is recommended for high-quality long reads (70-100bp). The SAM files were converted to BAM output which were subsequently sorted and indexed, and checked for the quality and mapping percentages per scaffold (S3 Table). The SAM files were then used to perform a SNP calling in Stacks (v.1.35, [51]).

In order to further assess the robustness of our inferences, we applied another complementary pipeline to reconstruct the genetic relatedness of our samples. Specifically, our goal was to infer population structure with Principal Component Analysis (PCA) using a statistical method based on genotype probabilities, rather than fixed called entities. This approach has been shown to be suitable for low or variable sequencing depth [54]. We used the software ANGSD v.0.911 [55] to filter low quality data and calculate genotype posterior probabilities with an informative prior under the assumption of Hardy Weinberg Equilibrium (HWE). We estimated the covariance matrix between samples using ngsTools [56], which takes data uncertainty into account. From such matrix, principal components were calculated and plotted using custom R scripts. This demonstrates that our main findings are not biased by the way data was processed.

### Phylogenetic inferences and species delimitation

We used the allelic data (71 out of the 95 total number of individual specimens) generated by nextRAD sequencing to build a maximum likelihood (ML) phylogenetic tree. We excluded the samples (24x) showing low genotype quality to minimize biases that could potentially be introduced by missing data (S1 File). The phylogenetic reconstruction was carried out in RAxML (v.7.2.8, [57]) using the GTR substitution model and GTRGAMMA as the GAMMA model of rate heterogeneity, with 1,000 bootstrap replicates and visualized in FigTree v.1.4.2 (http://tree.bio.ed.ac.uk/software/figtree).

### Population structure, admixture and evolutionary history

Several approaches were used to evaluate the genetic structure among populations within the *B*. *tabaci* species complex. A Principal Component Analysis (PCA), based on allelic data across all 71 whitefly samples was conducted using the SNPRelate R package [58]. ADMIXTURE (v.1.3.0 [59]) was performed on the whole dataset to estimate the genetic ancestry of each sample. This tool is based on a maximum likelihood approach which provides an estimate of the number of genetic clusters and the proportion of derived alleles in one sample from each of the K populations. The program was run multiple times, varying the values of K from 2 to 10. A cross-validation test was performed to determine the optimal value of K. An ABBA-BABA test also known as *D-*statistics was performed in ANGSD (v.0.911, [55]) in order to test for introgression between the two most invasive species MED and MEAM1 and the non-invader IO using the AUS species as an outgroup. The test compares the number of tree topologies of ABBA and BABA patterns. In absence of introgression, the number of ABBA and BABA trees should be equal and the expected value of Patterson’s D-statistic is zero.

The values of D-statistic that are above zero, correspond to a higher number of ABBA patterns, whereas negative values mean a higher frequency of BABA topologies. The significance of these D-statistic values is determined by the corresponding Z-scores, which are calculated in ANGSD with a jackknife procedure. An absolute value of the Z-score ≥ 3 is often used as a cut-off value. FineRADstructure, a software specifically designed for population inference from RADseq data, available at <https://github.com/millanek/fineRADstructure>, was used to investigate the genetic structure at the population level within the *B*. *tabaci* invasive species. The package includes RADpainter, a program designed to infer the co-ancestry matrix and estimate the number of populations within the dataset. The input file used was a haplotype matrix of our unmapped data (all 71 samples across species) generated by the *Populations* program in Stacks v. 1.35 (v1.35 [51]). Then, the individuals were assigned to populations and the phylogenetic tree was built using the fineSTRUCTURE MCMC clustering algorithm. *TreeMix* [60] was used to infer the history of population splits and admixtures, allowing up to ten migration events. This method constructs a bifurcating tree of populations using 100 bootstrap replicates. It, then, identifies potential episodes of gene flow from the residual covariance matrix.

## Results

### Data summary

#### nextRAD sequencing

A total of 95 samples were used to prepare the nextRAD libraries for sequencing and generated 49 million dual-indexed 110bp reads. Samples were filtered by read quality i.e. Phred score ≥ 20 and depth of coverage ≥ 3. A final set of 71 specimens were used in subsequent analyses and the remaining 24 samples were discarded due to low quality. The mean depth of coverage for each individual varied from 6X to 18X (Table 1, S1 Fig).

**Table 1:** Summary statistics of nextRAD sequencing output data for each *B*. *tabaci* populations. The species were initially genotyped using mtCOI sequencing. The raw data was filtered by quality and mapped against potential endosymbiont genomes. The filtered reads were then fed to the *de novo SNP* calling pipelines.

Country	Locality	Species	Raw reads	Filtered reads	% Used reads	n
Spain	Almeria	MED	6,339,910	3,661,150	57.74	6
Greece	Heraklion	MED	1,515,190	925,732	61.09	3
Croatia	Split	MED	1,327,720	946,094	71.25	3
Réunion	St Gilles	MED	935,916	711,348	76	2
France	Toulouges	MED	1,930,830	1,298,260	67.23	3
USA	Arizona	MED	4,892,870	2,276,580	46.52	6
Burkina Faso	Ouagadougou	MED	1,140,330	765,729	67.14	2
Burkina Faso	Sapone	MED	845,316	604,261	71.48	2
Israel	Tamra	MEAM1	1,579,040	930,447	58.92	2
Italy	Sicily	MEAM1	1,567,860	1,409,440	89.89	3
Turkmenistan	Ashgabad	MEAM1	2,786,060	2,191,440	78.65	3
Trinidad	Los Banos	MEAM1	486,529	476,864	98.01	3
Sudan	Gezira	MEAM1	1,808,650	1,425,470	78.81	3
Iran	Kerman	MEAM1	2,904,240	1,885,130	64.9	3
USA	Texas	MEAM1	2,640,490	2,054,130	77.79	3
Spain	Malaga	MEAM1	2,492,680	1,771,690	71.07	3
Réunion	St Gilles	MEAM1	5,122,320	3,316,390	64.74	9
Peru	Cañete Valley	MEAM2	1,943,380	1,696,340	87.72	3
Réunion	St Gilles	IO	3,697,650	2,770,960	74.93	6
Australia	Bundaberg	AUS	1,832,112	1,065,129	58.13	3

#### Mapping quality

The reads were aligned to the *B*. *tabaci* MEAM1 and MED genomes [23, 24]. Overall, the mapping percentage to the MEAM1 genome reference was above 80% across all samples except one sample from Sudan (78%) which is most likely caused by the low depth of sequencing and the DNA quality for this specimen. The mean average mapping percentage for the three major species considered in this study, was 89.96% for MED, 92.46% for MEAM1 and 88.57% for IO. The mapping to the MED genome showed similar results with average mapping percentages of 83.76 (IO), 87.57 (MED) and 84.65% (MEAM1) (S3 Table).

#### SNP calling

We conducted the SNP calling twice, first using a *de novo* approach (S2 Table), then using mapped reads to the two *B*. *tabaci* reference genomes available for MED and MEAM1) (S3 Table). The *de novo* SNP calling generated a total number of 38,041 SNPs from 71 individuals sampled in 17 countries. The number of SNPs identified when the reads were mapped in Stacks, to the MED and MEAM1 genomes were respectively 27,468 and 36,757 SNPs which are consistent with the *de novo* assembly findings. The subsequent population genomic analyses were performed using the three above-mentioned scenarios and gave consistent results, however, we are reporting the findings derived from the *de novo* SNP pipeline because it generated the highest number of SNPs and there was no requirement to rely on a functional annotation to identify specific genes or regions in the genome.

### Species delimitation

**Fig 2:**
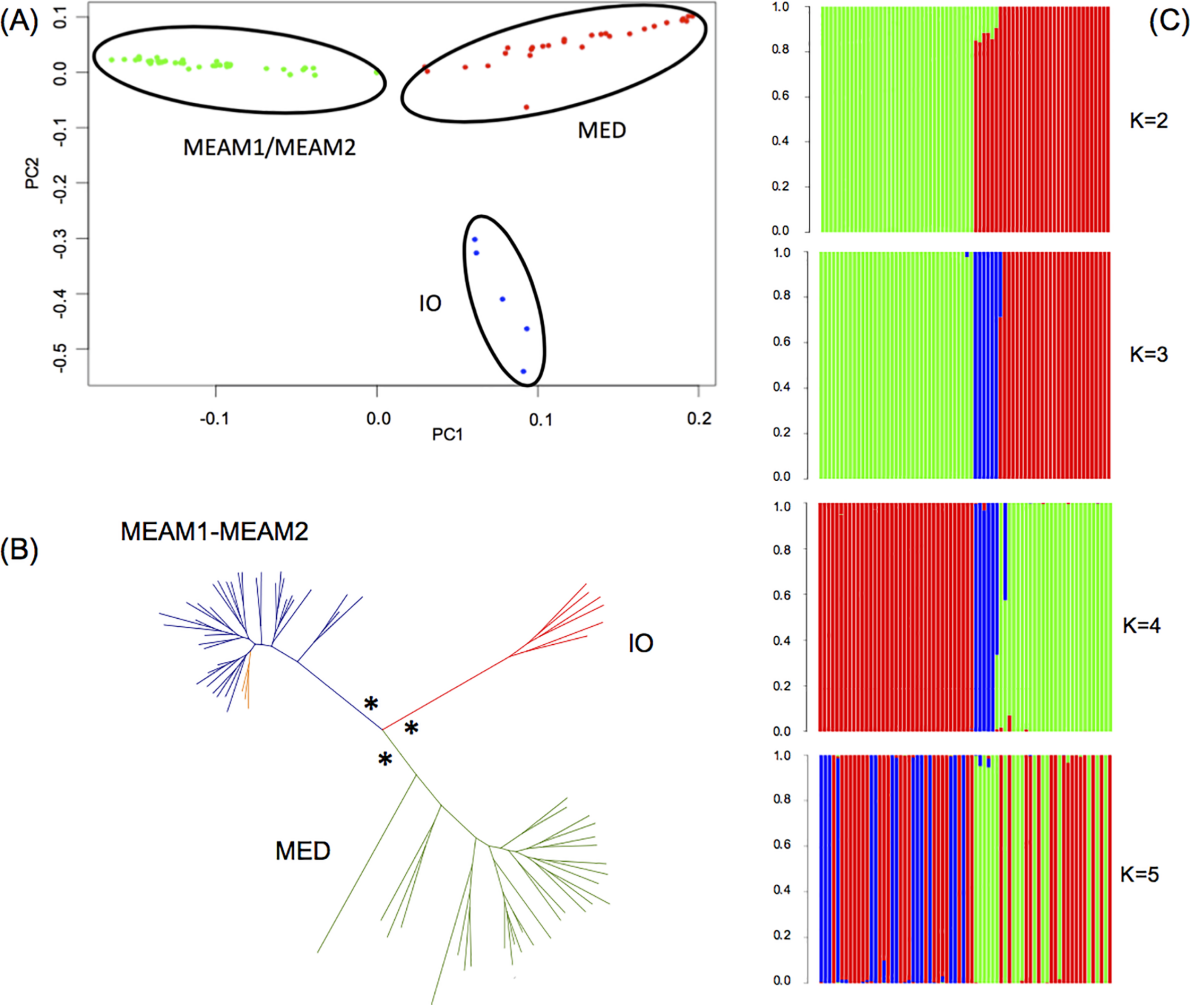
Interspecies relationships within the *B*. *tabaci* invasive clade. **(A)** Principal Component Analysis of 38,041 SNPs in 71 individual specimens. **(B)** Maximum likelihood phylogenetic tree constructed from concatenating 38,041 SNPs in 71 *B*. *tabaci* samples. The individuals highlighted in orange within the MEAM1 clade were genotyped as MEAM2 using mitochondrial DNA but could not be distinguished as a different species using genome-wide SNPs. (*) 100% bootstrap values. **(C)** ADMIXTURE analysis performed to estimate the optimal number of clusters (*k*) using the same set of SNPs as in the PCA. At the optimal K value of 3, the analysis reveals 3 genetic clusters corresponding to the species MED (red), MEAM1 (green) and IO (blue).

The Principal Component Analysis (PCA) shows that three of the four species, MED, MEAM1 and IO formed discrete clusters, the fourth, MEAM2, fell entirely within MEAM1 suggesting it may not be a separate species (Fig 2A, S3 Fig). Genome-wide SNPs were used to build a phylogeny. The individual-based maximum likelihood (ML) tree (Fig 2C) recovered three monophyletic clades with 100% bootstrap support. These clades correspond to MED, MEAM1 and IO; MEAM2 individuals were not phylogenetically distinct from MEAM1 (Fig 2C, S1 Table) supporting the results from the PCA (Fig 2A). The admixture plot (Fig 2D) revealed K = 3 as the most plausible scenario. A cross-validation test was performed, showing the optimal value of K = 3 (S2 Fig). The resulting clusters were consistent with the phylogeny and PCA results and as a consequence, for all future analyses, MEAM2 was considered synonymous with MEAM1.

### Admixture and signatures of recent gene flow

**Fig 3:**
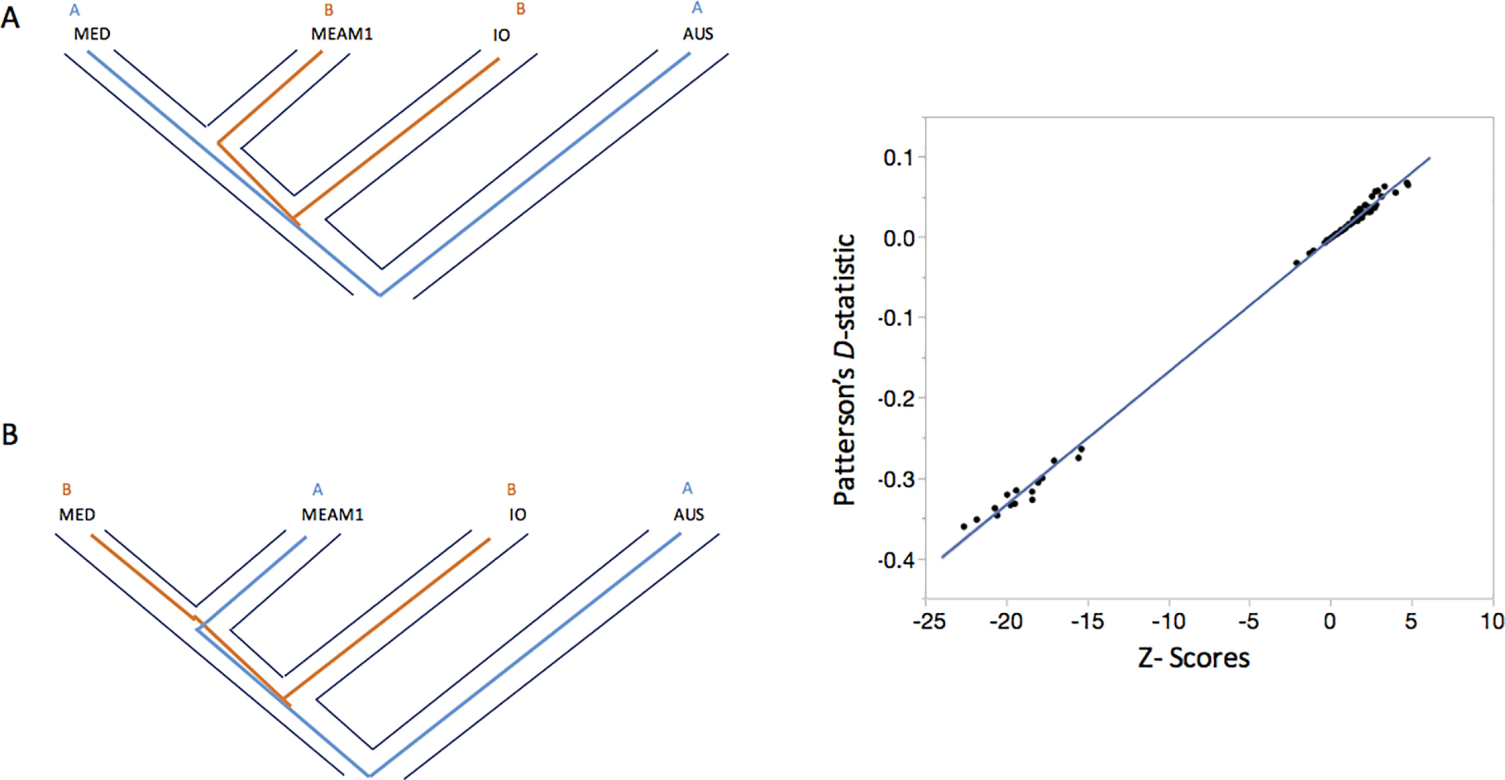
ABBA-BABA test of introgression. **(A)** ABBA pattern showing gene flow between MEAM1 and IO (red line) with AUS used as an outgroup. **(B)** BABA pattern showing gene flow between MED and IO (red line). **(C)**Plot showing the Z-scores to test the significance of the D-statistic test values.

The ABBA-BABA introgression test (also known as D-statistic) was performed to identify patterns of introgression between *B*. *tabaci* cryptic species MED, MEAM1 and IO using the *B*. *tabaci* AUS species as an outgroup (Fig 3). Here, the ABBA pattern, refers to possible introgression between MEAM1 and IO (Fig 3A) and the BABA to introgression between MED and IO (Fig 3B). Fig 3C shows the distribution of the Z-scores for all D-statistics values which were subsequently filtered according to the significance cut-off value (|Z-score| ≥ 3). The analysis of D-statistic values shows strong signals of introgression between MEAM1 and IO which is consistent with the ADMIXTURE analysis (Fig 2D). The D-statistic test also provides evidence that there is also introgression between MED and IO.

**Fig 4:**
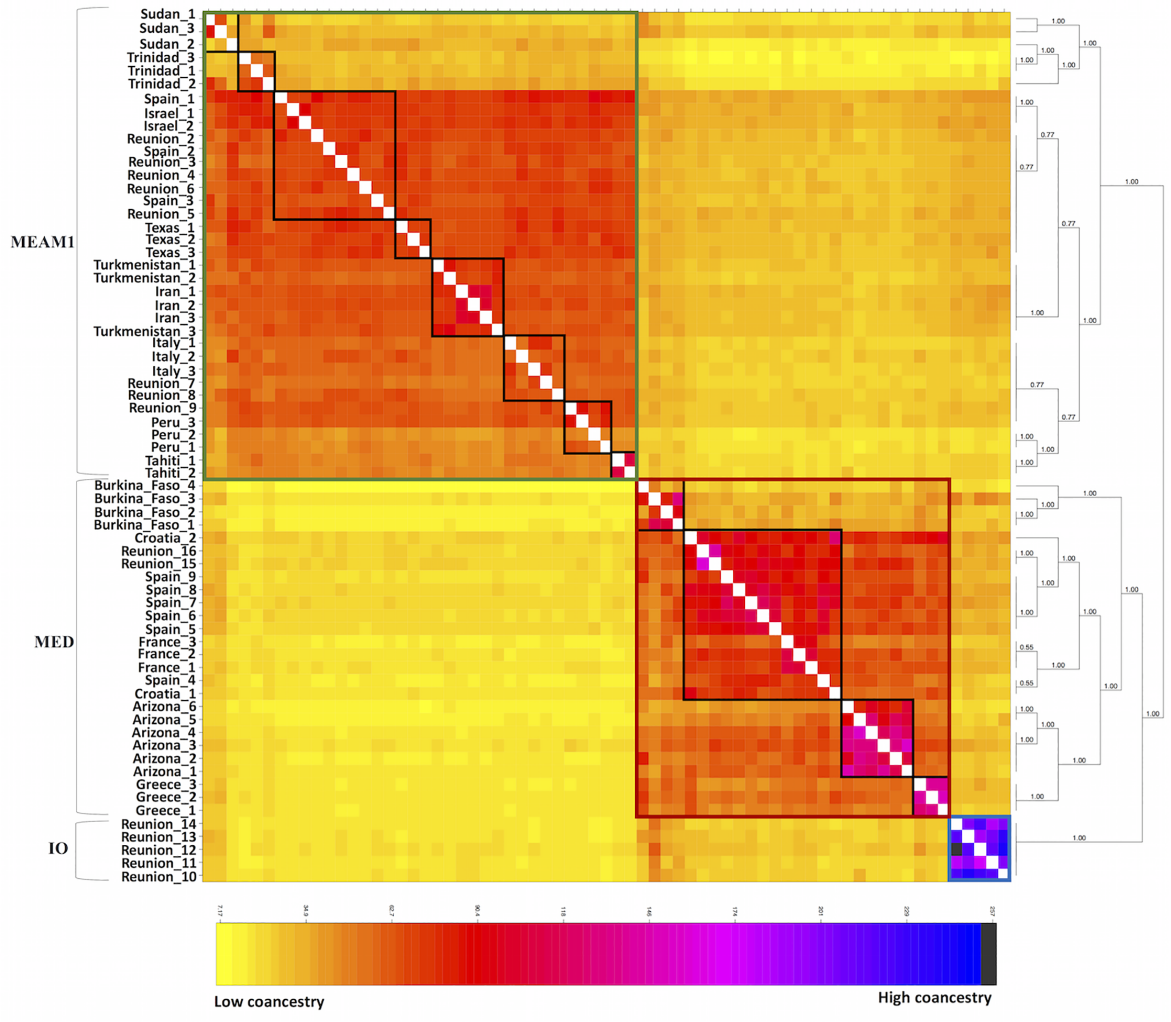
Coancestry heat map of the *B*. *tabaci* populations. The analysis conducted in FineRADSTRUCTURE identified three major clusters corresponding to the three *B*. *tabaci* species, (top left to bottom right MEAM1, MED and IO) across the dataset. The phylogenetic tree shows clustering by species and by geographical distribution within each species.

The clustered coancestry heat map, generated with FineRADstructure using genome-wide SNPs, also supports the existence of the three species, i.e. MED, MEAM1 and IO, with MEAM2 being part of MEAM1 (Fig 4). This analysis identified the single population in our dataset, within IO, had a high level of intrapopulation coancestry and this is most likely explained by the higher degree of isolation of this population from Reunion Island. The heat map showed that within the seven MED populations, three populations were clearly identified (Burkina Faso, Greece and Arizona), whereas the remaining four (France, Spain, Croatia and Reunion) formed a cluster, denoting gene flow within and between the Mediterranean Basin and Reunion Island. In the case of the eight MEAM1 populations included in the analysis, we identified four populations relating to Sudan, Trinidad, and Tahiti and Texas and three more complex population clusters. The first cluster includes Italy and Reunion, the second one harbors Spain, Israel and Reunion and the third, Iran and Turkmenistan. These two clusters reveal signatures of gene flow between Reunion and the Mediterranean Basin which is similar to patterns observed in populations within MED. The population from Peru, putatively labelled as MEAM2 is identified in this analysis as part of MEAM1 which further supports that MEAM2 is synonymous to MEAM1.

### Demographic history

**Fig 5:**
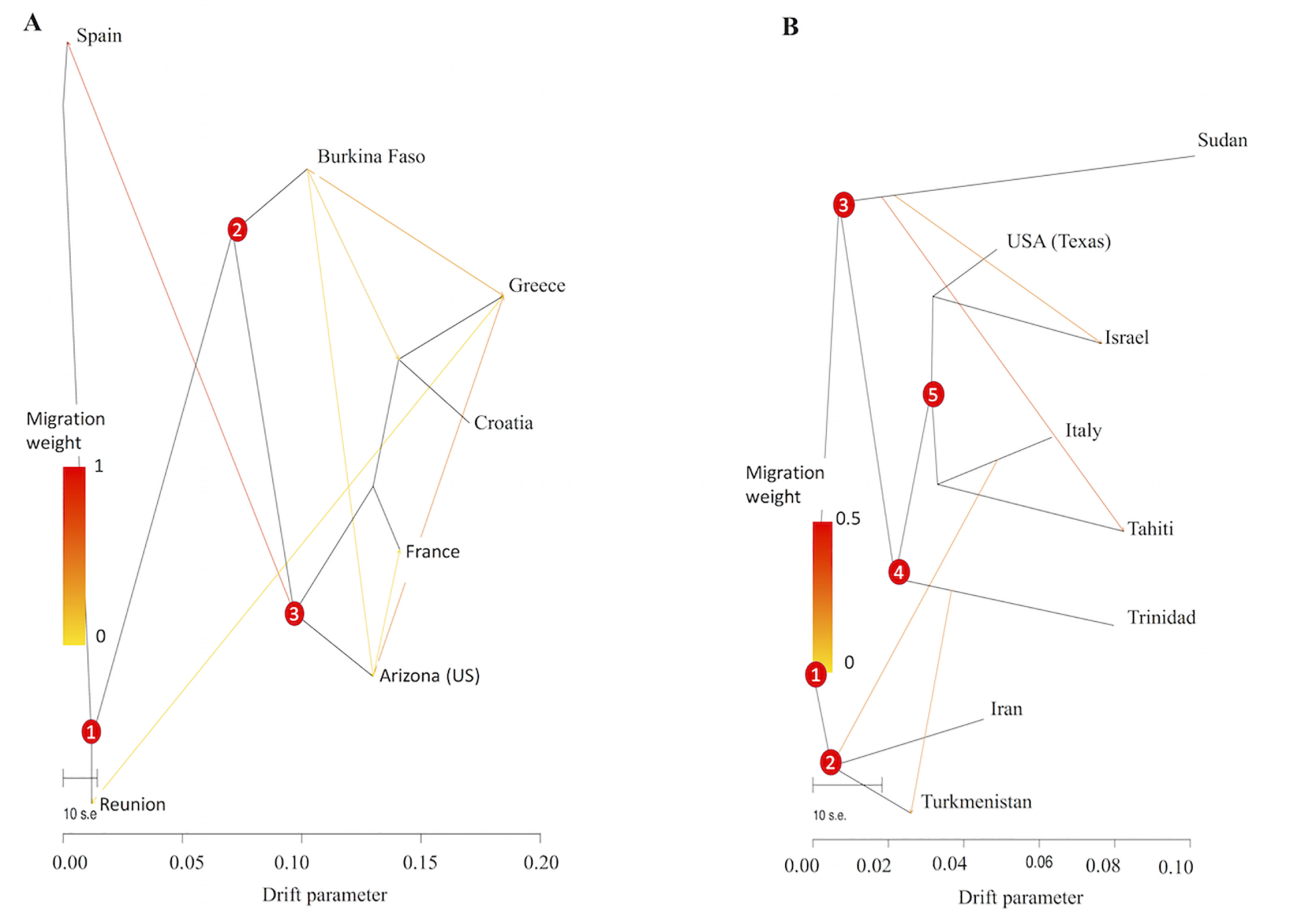
Demographic history of MED and MEAM1 populations. Inferred ML tree of MED **(A)** and MEAM1 **(B)** populations using *Treemix* [60]. The migration edges depicted by arrows show the gene flow direction. The drift parameter is proportional to *N*_e_ generations (*N*_e_: effective population size).

To further investigate admixture signals in the global invaders, MEAM1 and MED, we ran *TreeMix* [59] to generate a graph that best captures the relationships and infer the history of population splits and gene flow between populations based on the residual covariance matrices (S4 Fig). We constructed a bifurcating tree of seven populations for MED and eight populations for MEAM1, and examined the residual covariance matrix to identify pairs of populations that showed high levels of mixing (Fig 5). The tree for MED populations (Fig 5A) suggested divergence from an inferred ancestral population (1) into three lineages of Spain, proto-African (2) and Réunion. The proto-African lineage then diverged to give an African lineage and the contemporary invasive lineage (3) which gave rise to all invasive populations. The migration edges for MED (Fig 5A) showed strong gene flow between the invasive lineage and Spain that further pinpoint contemporary Spanish invasive populations. The population-based tree for MEAM1 (Fig 5B) supported divergence from an inferred ancestor (1) to the non-invasive Central Asia/Asia Minor lineage (2), and the invasive lineage (3). The migration edges for MEAM1 revealed signatures of admixture between populations from Israel and Italy. Other strong migration routes were depicted going from Trinidad to Reunion and from Reunion to Turkmenistan.

## Discussion

Studies focusing on the evolutionary ecology of the *B*. *tabaci* species complex have been undermined by the inability to obtain DNA material suitable for NGS experiments. Our study bypasses these limitations by relying on a novel and efficient RADseq protocol (nextRAD) that allowed us to obtain valuable information on a genome-wide scale from single individual whiteflies. This approach allowed us to generate a dense array of genome-wide SNPs, and therefore made it possible to tackle various questions that could not be addressed previously based on limited nuclear and mitochondrial markers. Our analysis identified 38,041 SNPs generated from the nuclear genome. These SNPs were used to build a phylogenetic tree, showing a topology, consistent with previous mtCOI studies, with the exception of the status of MEAM2. This strongly supports the species status of MED, MEAM1, IO and that MEAM2 is not a species, but rather is synonymous with MEAM1. Moreover, Tay et. al. 2017 [61] using comparative mitogenomics, showed that MEAM2 is not a real species but rather a pseudogene artifact of MEAM1. These findings are strengthened by the admixture analysis which also shows interspecies hybridization patterns. These patterns were further confirmed by an ABBA-BABA test which identified signatures of introgression between MEAM1 and IO confirming previous studies reporting gene flow between IO and MEAM1 in the field in Réunion Island [62]. Furthermore, evidence of incomplete mating isolation among the more closely related members of the complex where mtCOI diverge by ≤ 7% has been repeatedly demonstrated [63, 64]. Our results also show evidence of introgression between IO and MED in Réunion Island which had not been detected previously through the use of microsatellite DNA markers [20].

Our analysis of genome-wide SNPs to explore patterns of genetic mixing between populations of the same invasive species within the *B*. *tabaci* complex that were collected from various geographical localities worldwide enabled us to make inferences about migration events between these populations. In the case of MED, the genetic mixing analysis conducted using *Treemix* showed that the Sub-Saharan African population (Burkina Faso) is ancestral indicating that MED evolved in Sub-Saharan Africa before spreading to the Mediterranean Basin and supports mitochondrial DNA studies [12,15, 21]. Moreover, the Sub-Saharan African population from Burkina Faso is phenotypically distinct from those in the Mediterranean region in that it has retained the capacity to induce Silverleafing in squash [65]. This ability is also retained in MEAM1 and IO and suggests that the Silverleafing phenotype is an ancestral feature of the invasive clade. Our results also depicted a number of strong signals of migration between geographically quite separate populations. In the case of MED, we have several examples including gene flow between Sub-Saharan Africa (Burkina Faso) and the Mediterranean region (France, Croatia and Greece), between Burkina Faso and USA (Arizona) and another migration event from Arizona (USA) to Greece. This is best explained by the role played by the trade in ornamental plants [66, 67].

In the case of Réunion, Thierry *et al*. (2015) [68] concluded that the recent invasion by MED of Réunion Island involved genotypes that originated in both the eastern and western parts of the Mediterranean Basin. Our results support this as they show both a strong pattern of gene flow between Greece and Réunion Island and between Réunion and Spain. MEAM1 shows a similar set of signals that support migration. The analysis of genetic mixing of populations within the MEAM1 species positions populations from Iran and Turkmenistan as ancestral to the rest, a finding supported by historical records which inferred that MEAM1 originally spread from the Middle East–Asia Minor region [16].

Our results revealed a migration route from Israel to Italy. Another migration event was identified from Trinidad to Réunion Island which might be explained by the ornamental trade. Further sampling is required to identify intermediate steps along this particular migration route. An intriguing migration event from a more recent or derived population (Réunion) to an ancestral population (Turkmenistan) was also depicted. Here, rather than looking at invasion as a unidirectional process based on detections of novel outbreaks, our analysis enables us to some extent to see that the process of invasion is ongoing and bidirectional between the home and invaded ranges. Our data provide evidence of repeated invasion events in both directions that are resulting in repeated exchanges of new genetic information. This process may lead to the gradual accumulation of traits that favor invasion (e.g. insecticide resistance genes) and subsequently increase the pest status of the invader [69]. The inclusion of more populations within MED and MEAM1 across the invaded range is likely to uncover further patterns of gene flow connectedness and demographic scenarios. Our analysis sets the foundation for further exploring the global invasion history of *B*. *tabaci* invasive species.

## Supporting information

S1 File Additional bioinformatic data analyses. (DOCX)

S1 Fig Mean depth of coverage of nextRAD samples. (TIFF)

S2 Fig Cross-validation error plot (admixture analysis). (TIFF)

S3 Fig Principal component analysis (PCA) plots generated using ANGSD/ngsTools. (TIFF)

S4 Fig Covariance matrices (Treemix analysis). (TIFF)

S1 Table Genome-wide SNP’s species delimitation analysis. (PDF)

S2 Table Additional data summary. (XLSX)

S3 Table SNP calling statistics. (XLSX)
