# Supplementary material for: Genome-wide analyses of the Bemisia tabaci species complex reveal contrasting patterns of admixture and complex demographic histories
Source: PLoS One. 2018 Jan 24;13(1):e0190555. doi: 10.1371/journal.pone.0190555 (PMC5783331; doi:10.1371/journal.pone.0190555)
Supplement: S1 File — (DOCX) [file pone.0190555.s001.docx]

**Experimental Procedures**

**Quality control.** The quality of the fastq sequences was assessed using FastQC (http://www.bioinformatics.babraham.ac.uk/projects/fastqc) which provides a report on quality scores per sequence, N content, GC content and sequence duplication levels. Based on these reports, a trimming by quality (Phred quality score < 20), to a length of 101 bp, was done in Trimmomatic [1] using the following command:

***“java*** *–jar trimmomatic-0.33.jar SE –phred33 input.fq output.fq AVGQUAL:15 LEADING:3 TRAILING:3 MINLEN:101 CROP:101”.*

**SNP calling.** The fastq sequences were fed to the *Stacks* (v1.35, [2]) pipeline for SNP calling, using the following commands:

- *ustacks -t fastq -f ./1.fq -o ./ -r -m 2 -M 2 -N 4 -i 1*
- *cstacks -b 1 -o ./ -n 2 -s ./8-1 -s ./8-3 -s ./8-5 -s ./9-4 -s ./9-5 -s ./9-6 -s ./19-1 -s ./19-2 -s ./19-3 -s ./19-5 -s ./19-6 -s ./23-3 -s ./23-5 -s ./23-6 -s ./26-4 -s ./26-5 -s ./29-3 -s ./30-1 -s ./30-2 -s ./30-3 -s ./31-1 -s ./31-2 -s ./31-3 -s ./31-4 -s ./31-5 -s ./31-6 -s ./33-2 -s ./33-3 -s ./33-5 -s ./42-2 -s ./42-3 -s ./44-1 -s ./44-4 -s ./44-5 -s ./47-1 -s ./47-2 -s ./47-4 -s ./83-2 -s ./83-3 -s ./83-4 -s ./83-5 -s ./84-1 -s ./84-2 -s ./84-3 -s ./84-4 -s ./84-5 -s ./84-6 -s ./89-1 -s ./89-3 -s ./92-1 -s ./92-3 -s ./92-5 -s ./93-1 -s ./93-2 -s ./93-3 -s ./93-4 -s ./93-5 -s ./93-6 -s ./108-3 -s ./108-4 -s ./108-5 -s ./109-1 -s ./109-5 -s ./109-6 -s ./112-2 -s ./112-3 -s ./115-2 -s ./115-3 -s ./129-1 -s ./129-2 -s ./129-3*
- *sstacks -b 1 -c batch_1 -o ./ -s 1*
- *populations –b 1 –M ./map.txt –r 1 –p 1 –P ./*

The last step in the *Stacks* pipeline (*populations*) generated summary statistics output files including a vcf file, which was fed to VCFtools [3] to extract the genotypes and the read depth per site for every individual sample in the dataset.

**Species delimitation.** Based on genome-wide SNPs, the interspecies differences were further explored using the species delimitation plugin implemented in Geneious v9.0.5 [4] (Biomatters Ltd., Auckland, New Zealand). The Geneious Species Delimitation plugin allows the computation of the Rosenberg index P*_(AB)_*, which represents the probability that species A represented by a set of sequences, in a clade of (a + b) sequences, will be reciprocally monophyletic with the remaining b sequences under the null model of random coalescence.

**References**

1. Bolger AM, Lohse M, Usadel B. Trimmomatic: a flexible trimmer for Illumina sequence data. Bioinformatics. 2014 Apr 1: btu170.
2. Catchen J, Hohenlohe PA, Bassham S, Amores A, Cresko WA. Stacks: an analysis tool set for population genomics. Molecular ecology. 2013 Jun 1;22(11):3124-40.
3. Danecek P, Auton A, Abecasis G, Albers CA, Banks E, DePristo MA, Handsaker RE, Lunter G, Marth GT, Sherry ST, McVean G. The variant call format and VCFtools. Bioinformatics. 2011 Aug 1;27(15):2156-8.
4. Masters BC, Fan V, Ross HA. Species delimitation–a geneious plugin for the exploration of species boundaries. Molecular Ecology Resources. 2011 Jan 1;11(1):154-7.

**S1 Table.** Genome-wide SNP’s species delimitation analysis of *B. tabaci* cryptic species, using the Species Delimitation plugin (Geneious v8.0).

| **Species** | **Closest Species** | **Intra Dist.** | **Inter Dist** | **Intra/ Inter** | **P_ID_(Strict)** | **P_ID_(Liberal)** | **Av(MRCA-tips)** | **P (Randomly Distinct)** | **Rosenberg's P_(AB)_** |
| --- | --- | --- | --- | --- | --- | --- | --- | --- | --- |
| IO | MED | 0.951 | 2.351 | 0.4 | 0.66 (0.54, 0.79) | 0.90 (0.80, 1.0) | 0.6545 | 0.68 | 4.50E-08 |
| MED | IO | 0.833 | 2.351 | 0.35 | 0.88 (0.83, 0.94) | 0.97 (0.94, 0.99) | 1.0627 | 0.98 | 4.50E-08 |
| MEAM1 | IO | 0.842 | 3.111 | 0.27 | 0.91 (0.85, 0.96) | 0.97 (0.94, 1.00) | 1.0315 | 1 | 1.00E-24 |

Intra Dist.: average pairwise tree distance among members of a predefined clade

Inter Dist.: average pairwise tree distance between members of the group of interest and its sister taxa

Intra/Inter: The ratio of Intra Dist to Inter Dist

P_ID_(Strict): mean probability, with a 95% confidence interval (CI) for a prediction of making a correct identification of an unknown specimen being found only in the group of interest

P_ID_(Liberal): mean probability, with a 95% confidence interval (CI) for a prediction of making a correct identification of an unknown specimen being sister to or within the group of interest

Av_(MRCA-tips)_: mean distance between the most recent common ancestor of the species and its members

P _(Randomly Distinct)_: probability that a clade has the observed degree of distinctiveness

Rosenberg’s P_(AB)_: Reciprocal monophyly (H_0_: a monophyly is an outcome of random branching).

**S1 Fig.** Mean depth of coverage of nextRAD sequencing for individual specimen in the *B. tabaci* dataset.

K

Cross validation error

**S2 Fig.** Cross-validation error plot (admixture analysis). K=3 has the lowest cross-validation (CV) error value.

**S3 Fig.** Principal component analysis (PCA) plots generated using the ANGSD/ngsTools pipeline. (A) PCA of *B. tabaci* putative species (MED, MEAM1, MEAM2 and IO), (B) PCA showing three species (MED, MEAM1-MEAM2 and IO), (C) PCA of populations from MEAM1 species, (D) PCA of populations from MED species.


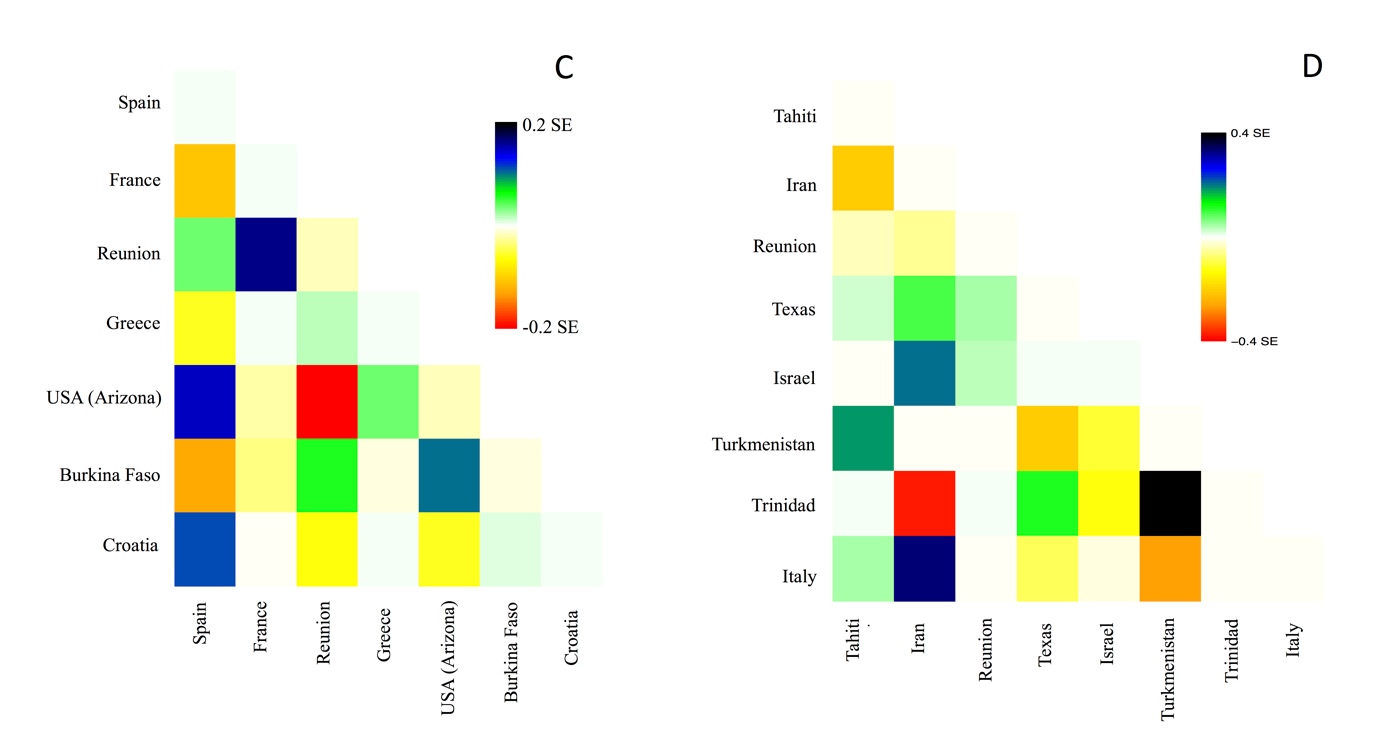


**S4 Fig.** The covariance matrix corresponding to the MED (S4C Fig) and MEAM1 (S4D Fig.) represent the residual plots corresponding to the phylogeny models for each of the two species. Residuals above zero represent populations that are more closely related to each other in the data than in the ML tree, and therefore are candidates for admixture events.
