# Supplementary figures and images for: Genome-wide analyses of the Bemisia tabaci species complex reveal contrasting patterns of admixture and complex demographic histories
Source: PLoS One. 2018 Jan 24;13(1):e0190555. doi: 10.1371/journal.pone.0190555 (PMC5783331; doi:10.1371/journal.pone.0190555)

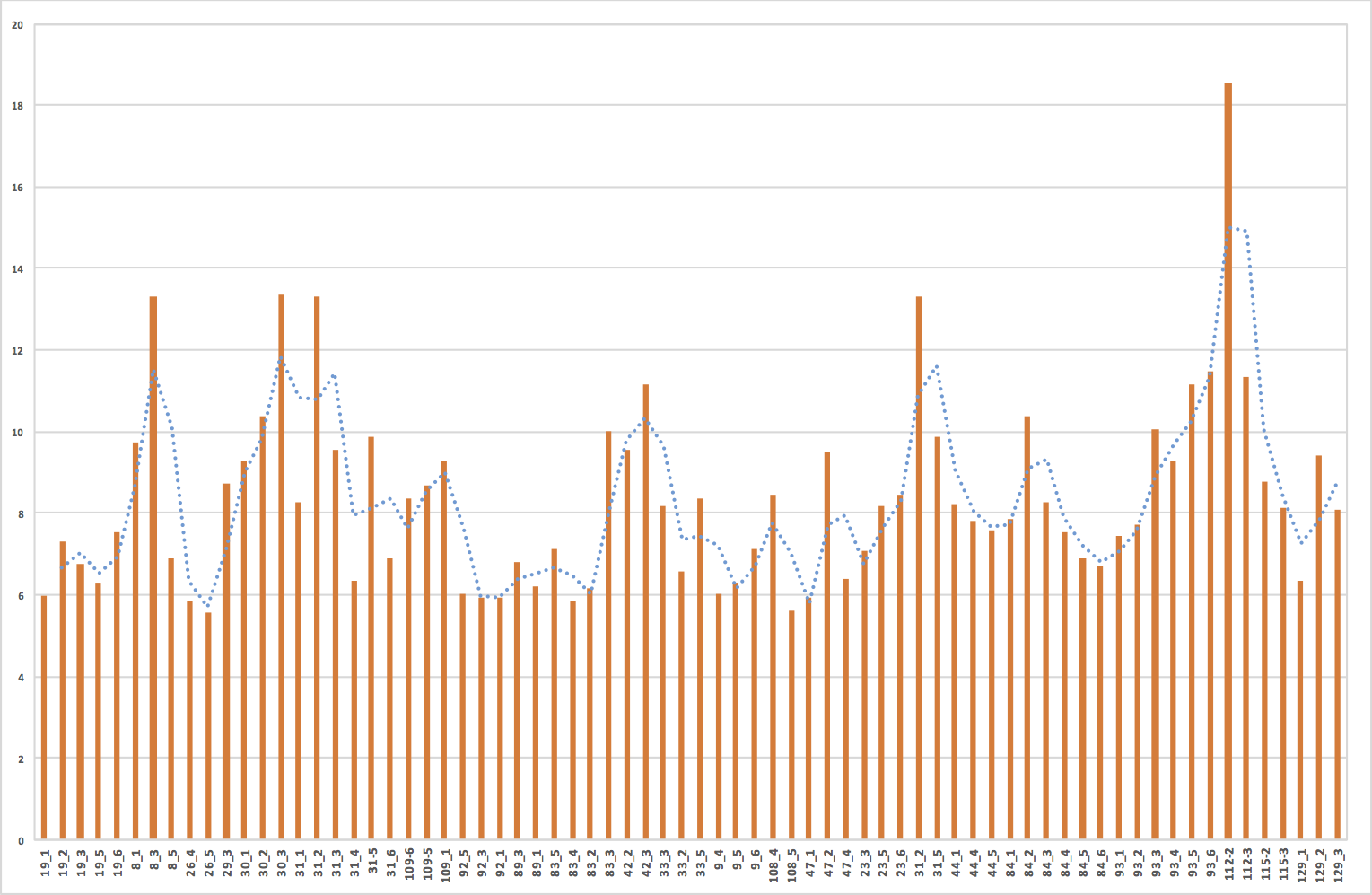

Supplement: S1 Fig — (TIFF) [file pone.0190555.s002.tiff]

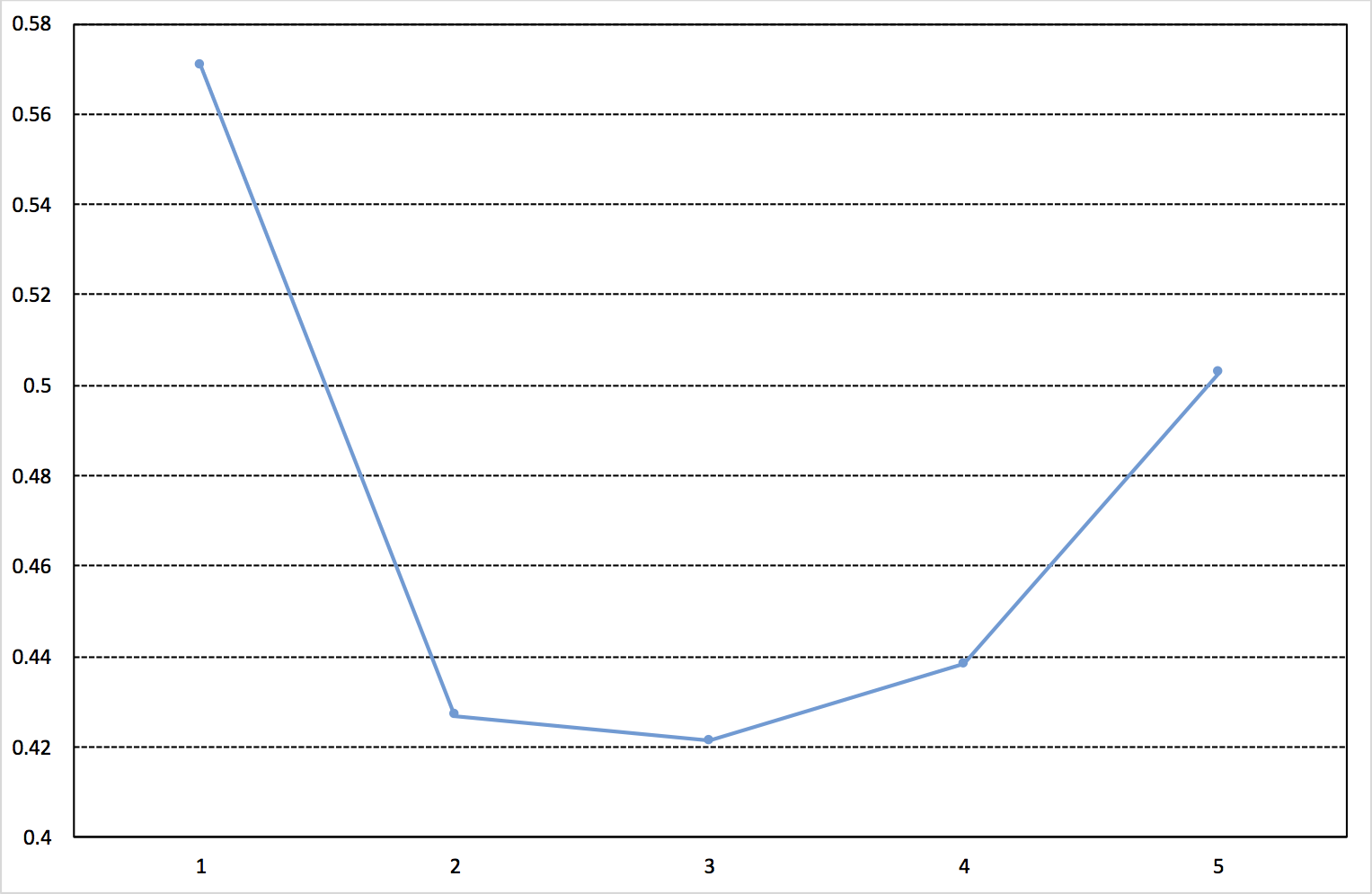

Supplement: S2 Fig — (TIFF) [file pone.0190555.s003.tiff]

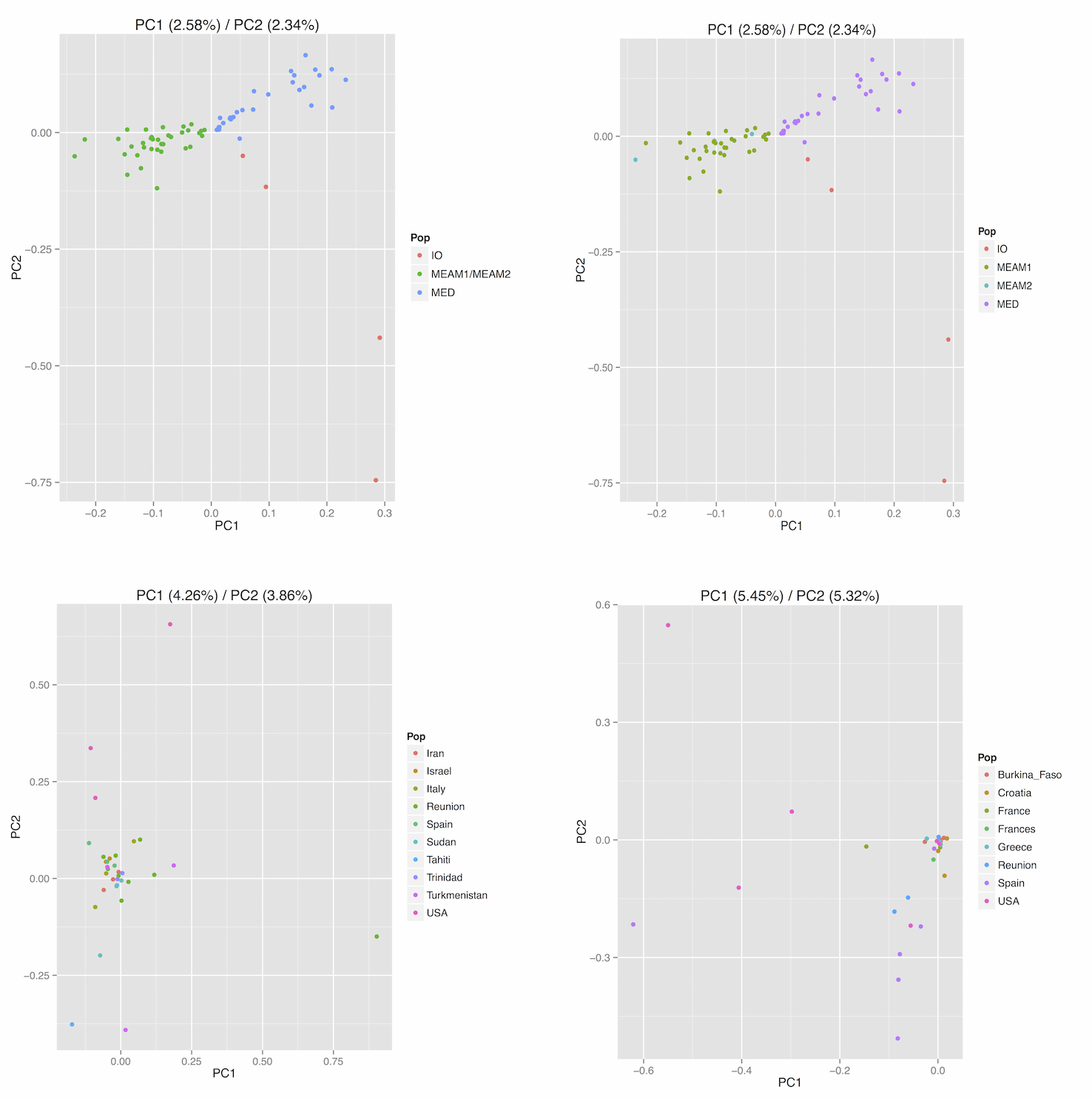

Supplement: S3 Fig — (TIFF) [file pone.0190555.s004.tiff]

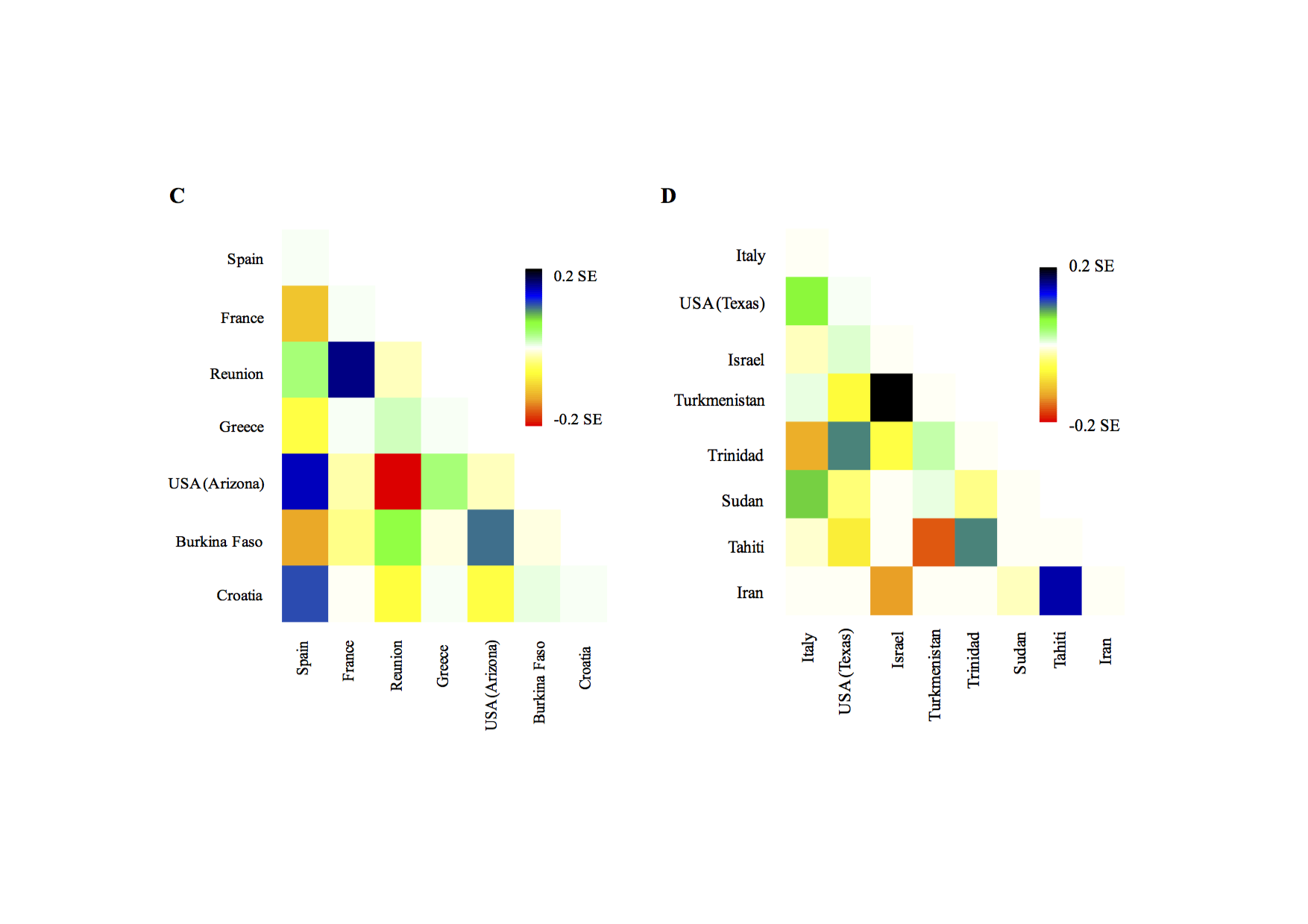

Supplement: S4 Fig — (TIFF) [file pone.0190555.s005.tiff]
