# Supplementary material for: Genome-wide analyses of the Bemisia tabaci species complex reveal contrasting patterns of admixture and complex demographic histories
Source: PLoS One. 2018 Jan 24;13(1):e0190555. doi: 10.1371/journal.pone.0190555 (PMC5783331; doi:10.1371/journal.pone.0190555)
Supplement: S1 Table — (PDF) [file pone.0190555.s006.pdf]

S1 Table. Genome-wide SNP's species delimitation analysis of *B. tabaci* cryptic species, using the Species Delimitation plugin (Geneious v8.0).

Species	Closest Species	Intra Dist.	Inter Dist	Intra/ Inter	P_{ID}(Strict)	P_{ID}(Liberal)	Av(MRCA-tips)	P (Randomly Distinct)	Rosenberg's $P_{(AB)}$
IO	MED	0.951	2.351	0.4	0.66 (0.54, 0.79)	0.90 (0.80, 1.0)	0.6545	0.68	4.50E-08
MED	IO	0.833	2.351	0.35	0.88 (0.83, 0.94)	0.97 (0.94, 0.99)	1.0627	0.98	4.50E-08
MEAM1	IO	0.842	3.111	0.27	0.91 (0.85, 0.96)	0.97 (0.94, 1.00)	1.0315	1	1.00E-24

Intra Dist.: average pairwise tree distance among members of a predefined clade
Inter Dist.: average pairwise tree distance between members of the group of interest and its sister taxa
Intra/Inter: The ratio of Intra Dist to Inter Dist
P_{ID}(Strict): mean probability, with a 95% confidence interval (CI) for a prediction of making a correct identification of an unknown specimen being found only in the group of interest
P_{ID}(Liberal): mean probability, with a 95% confidence interval (CI) for a prediction of making a correct identification of an unknown specimen being sister to or within the group of interest
$Av_{(MRCA\text{-}tips)}$: mean distance between the most recent common ancestor of the species and its members
$P_{(Randomly\ Distinct)}$: probability that a clade has the observed degree of distinctiveness
Rosenberg's $P_{(AB)}$: Reciprocal monophyly (H_0: a monophyly is an outcome of random branching
